# Effect of Pituitary-Target Gland Axis on RAAS in the Context of COVID-19

**DOI:** 10.7150/ijms.114924

**Published:** 2025-07-25

**Authors:** Baofeng Wu, Ru Li, Qinhao Liu, Shuqing Jin, Hongxia Wei, Ming Xu, Yi Zhang, Yunfeng Liu

**Affiliations:** 1 Department of Endocrinology, First Hospital of Shanxi Medical University, Taiyuan, 030001, China.; 2 First Clinical Medical College, Shanxi Medical University, Taiyuan, 030001, China.; 3 Department of Pharmacology, Shanxi Medical University, Taiyuan, 030001, China.; 4 Clinical Research Center of Endocrine and Metabolic Disease in Shanxi Medical University, Taiyuan, 030001, China.

**Keywords:** Pituitary gland, Renin-angiotensin-aldosterone system, Pituitary hormones, COVID-19, SARS-CoV-2

## Abstract

The pituitary gland is a very important endocrine gland in the human body. It secretes and releases many hormones crucial for controlling physiological processes, such as energy metabolism, human growth and development, and reproduction. The renin-angiotensin-aldosterone system regulates water and salt homeostasis, controlling blood pressure. Since the discovery of the renin-angiotensin-aldosterone system, exploring and studying its role in pathophysiology has never stopped, and patients have benefited from drug-based and clinical studies. This review focuses on the effects of the pituitary-target gland axis (pituitary-thyroid axis, pituitary-adrenal axis, pituitary-growth hormone axis, pituitary-gonadal axis) and some hormones secreted and stored by the pituitary gland on the RAAS. While considering that SARS-CoV-2 reinfection still occurs, we aim to provide new insights into water-electrolyte balance and blood pressure regulation.

## Introduction

The endocrine system primarily consists of endocrine glands (including the pituitary, thyroid, parathyroid, adrenal, and gonads), as well as endocrine tissues and cells found in various organs, such as the cardiovascular, gastrointestinal, renal, adipose tissue, and brain (especially the hypothalamus). The endocrine system is an important regulatory system of the body, complementing the nervous system in maintaining the balance and stability of the internal environment. It regulates the growth and development of the body, as well as various metabolic activities, and influences various behaviors. The hypothalamus-pituitary-target gland axis plays a vital role in the homeostasis of hormone secretion. The pituitary gland is a grey-red oval-shaped body located in the pituitary fossa of the pterygoid saddle at the base of the skull, which can be divided into two major parts: the adenohypophysis and the neurohypophysis. The adenohypophysis secretes growth hormone (GH), thyroid-stimulating hormone (TSH), adrenocorticotropic hormone (ACTH), and gonadotropin, while the neurohypophysis stores and releases antidiuretic hormone (vasopressin) and oxytocin, synthesized by neuroendocrine cells in the supraoptic nucleus and paraventricular nucleus of the hypothalamus 1, 2. The pituitary gland is the most important endocrine gland in the body, controlling the secretion of various hormones essential for metabolism, growth, development, and reproduction.

The renin-angiotensin-aldosterone system (RAAS) is vital in the body, maintaining plasma sodium concentration, arterial blood pressure, and extracellular fluid volume. Renin is a proteolytic enzyme synthesized, stored, and released by the juxtaglomerular cells that catalyzes the conversion of plasma angiotensinogen to angiotensin I (Ang I). Most of the renin is produced in the kidneys. Renin release is affected by the perfusion pressure of the glomerulus arteriole, the concentration of Na^+^ in the filtrate of the distal tubule flowing through the macula densa, and by renal sympathetic nerves 3, 4. Ang I generates angiotensin II (Ang II) under the action of the angiotensin converting enzyme (ACE), which induces vasodilation by binding to the Ang II receptor 1 (AT1R). In the proximal convoluted tubules of the kidney, Ang II may increase Na^+^-H^+^ exchange, thereby increasing sodium reabsorption and raising arterial pressure in patients. Ang II also acts on the adrenocortical globular zone, which stimulates aldosterone release, thereby affecting sodium reabsorption and potassium excretion in the distal convoluted tubules and collecting ducts of the renal unit 5.

**Figure 1:**
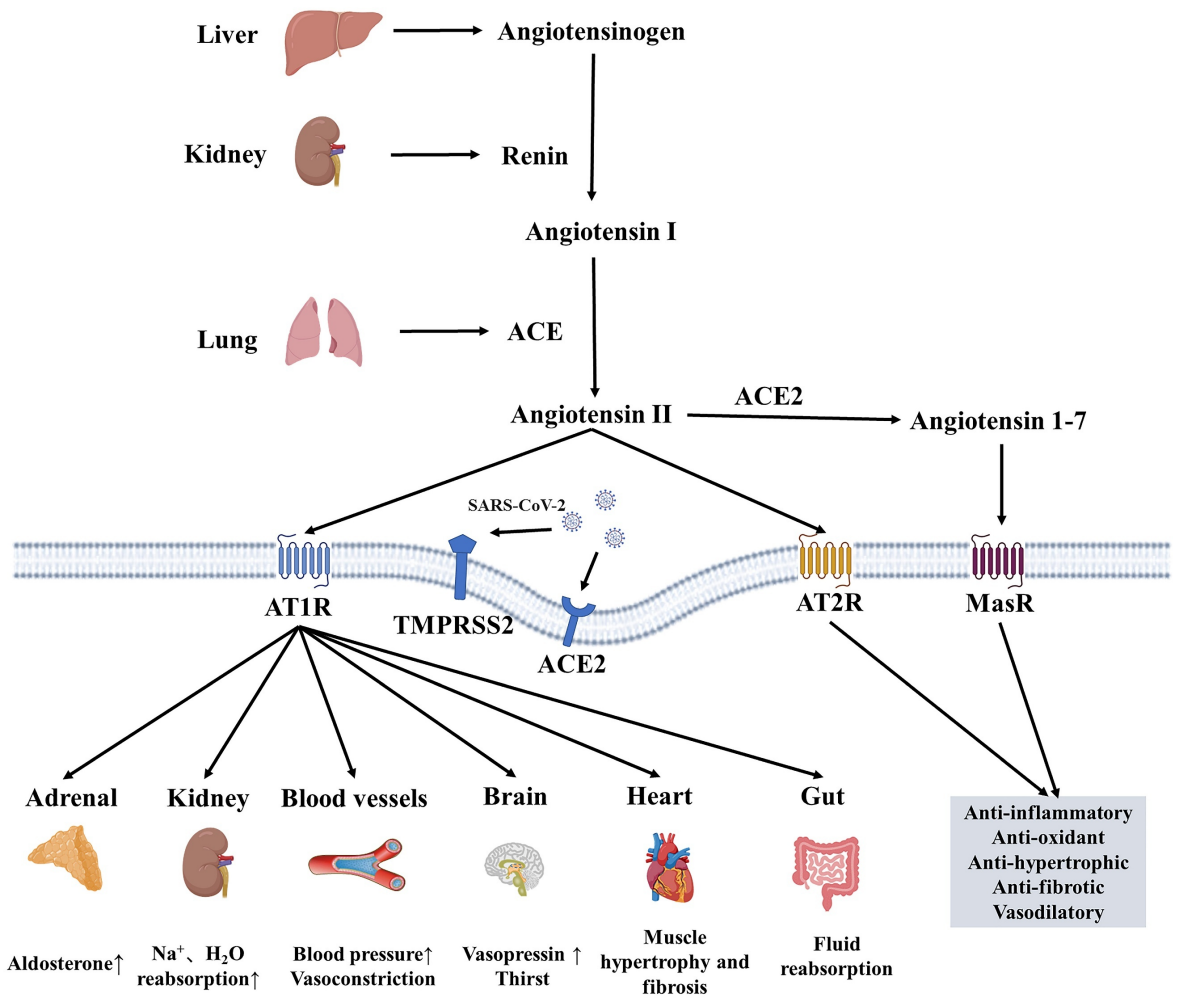
** The Renin-Angiotensin-Aldosterone-System and the influence of SARS-CoV-2 on it.** RAAS comprises classic ACE/Ang II/AT1 axis and non-classic ACE2/Ang(1-7)/Mas axis. ACE/Ang II/AT1 exerts its physiological effects through ATIR on many organs, such as the heart, brain and kidney, while ACE2/Ang(1-7)/Mas exerts antioxidant and anti-fibrotic effects. ACE2 and TMPRSS2 can mediate the internalization and infection of SARS-CoV-2, and reduce the protective ACE2/Ang(1-7)/Mas components, resulting in an imbalance of RAAS balance. ACE, angiotensin-converting enzyme; ACE2, angiotensin-converting enzyme 2; AT1R, angiotensin II receptor type 1; AT2R, angiotensin II receptor type 2; MasR, Mas receptor; TMPRSS2, transmembrane protease serines 2.

RAAS comprises the classical ACE/Ang II/AT1R axis and the non-classical ACE2/Ang(1-7)/Mas axis 6. The ACE2/Ang(1-7)/Mas axis antagonizes the ACE/Ang II/AT1R axis, becoming the primary regulatory mode of the RAAS system 6, 7. After SARS-CoV-2 infection, studies have found that ACE2, a receptor for SARS-CoV-2, mediates virus internalization and infection. However, SARS-CoV-2 binding to ACE2 downregulates ACE2 activity, leading to the accumulation of Ang II and excessive RAS activation 8, 9. Elevated Ang II leads to vasoconstriction, inflammation, cell differentiation and growth, endothelial dysfunction, the production of reactive oxygen species, and microthrombosis (**Figure 1**). Circulating ACE2 is a biomarker of COVID-19 mortality 10. A study following SARS-CoV-2 infection revealed that Ang II levels were higher in COVID-19 patients compared to controls, and a linear relationship existed between high Ang II levels and adverse clinical outcomes 11. Hypertension may increase the risk of severe or fatal COVID-19 in patients by 2.5 times, especially in older patients 12. Angiotensin-converting enzyme inhibitors (ACEIs)/ Angiotensin receptor blockers (ARBs) can help alleviate multi-organ damage caused by excessive activation of RAAS without aggravating SARS-CoV-2 infection and reduce all-cause mortality in hospitalized patients 13, 14.

RAAS plays a crucial role in maintaining water and electrolyte balance. Most previous studies have focused on the role of the RAAS in the occurrence and development of diseases, as well as its use as a target for drug treatment. In contrast, there have been few studies on the effect of endocrine-related factors on the RAAS. Secondly, considering endocrine glands such as the hypothalamus, pituitary, thyroid, adrenal, testes, and ovaries express ACE2^15^, the long-term effects of SARS-CoV-2 infection on these organs and the RAAS are not very clear. Therefore, this article describes the effects of pituitary and target gland hormones on RAAS from the perspective of the pituitary-target gland axis and explores the possible mechanisms. At the same time, combined with the potential long-term effects of SARS-CoV-2 infection, it offers new insights into maintaining water and electrolyte balance and regulating blood pressure.

## COVID-19 and the Pituitary gland

As mentioned earlier, ACE2 is expressed in various organs and tissues, allowing the infection to spread rapidly beyond the respiratory system to other organs. The SARS-CoV-2 virus may enter the brain through the blood-brain barrier via the nasopharyngeal epithelium or systemic vascular circulation 15. Although ACE2 expression is low in the pituitary, SARS-CoV-2 has a strong binding ability with ACE2, which means that the pituitary is more susceptible to direct damage by the virus 16. COVID-19 has been found to cause a variety of pituitary injuries, including pituitary apoplexy, hypophysitis, hypopituitarism, and arginine vasopressin deficiency 17-20. For example, among post-infection hypophysitis caused by COVID-19, studies have found that cases of post-infection hypophysitis are relatively rare; however, the onset age of COVID-19-related hypophysitis is lower than that of other types, and the incidence is higher in pediatric patients, especially adolescents 21. Interestingly, the most common pituitary disease after COVID-19 vaccination was hypophysitis. Secondly, due to the harmful effects of SARS-CoV-2 infection on the hypothalamus and pituitary gland, as well as the widespread use of high-dose corticosteroids for treatment, patients with COVID-19 may have an increased risk of long-term adrenal insufficiency and are more likely to experience long-term sequelae 22.

After SARS-CoV-2 infection, sequelae can last from one week to several months and may even persist for life; the World Health Organization (WHO) refers to these conditions as "Long COVID-19"^23^. SARS-CoV-2 is a neurotropic virus, and the central nervous system is also a common target of SARS-CoV-2. In COVID-19-related studies, compared to other central nervous system studies, less research has been conducted on the pituitary. Studies have found that insufficient secretion of adrenocorticotropic hormone and growth hormone may be related to the pathogenesis of the long-term syndrome associated with COVID-19^24^. These findings underscore the complexity of Long COVID-19 and emphasize the importance of investigating the endocrine system, particularly the pituitary gland, to comprehend and address COVID-19 complications. Focusing on and addressing the hormonal disorders in patients with Long COVID-19 may provide a new way for the management and care of the disease.

## Pituitary-thyroid axis and RAAS

Thyrotropin-releasing hormone (TRH) secreted by the hypothalamus stimulates the secretion of thyroid-stimulating hormone (TSH) by the anterior pituitary, which initiates the synthesis of thyroid hormone (TH) and its release from the thyroid gland through the TSH receptor (TSHR). TSHR belongs to the G protein-coupled receptor family, and its main expression site is the basolateral membrane of thyroid cells. TSHR is also expressed in the kidney and adrenal glands 25. TH plays a vital role in the growth and development of various tissues, including the kidneys. The kidney is involved in the metabolism and elimination of TH, and renal insufficiency produces various changes in the metabolism of TH and upstream hormones, such as an increased basal TSH value, alterations in the TSH circadian rhythm, and impaired renal clearance of TSH 26. The kidney is the site of renin release and angiotensin synthesis, and thyroid dysfunction may lead to abnormal RAAS function 27, 28.

Thyroid hormones influence the synthesis of angiotensinogen and other RAS components and ACE activity, and abnormal secretion of thyroid hormones can affect blood pressure. Hypothyroidism can manifest as decreased cardiac output and blood pressure, which is associated with a low plasma renin concentration and activity. This is because hypothyroidism renders the kidneys less sensitive to β-adrenergic stimulation, resulting in reduced renin gene expression and release, and ultimately, reduced RAAS activity 29. Hyperthyroidism leads to the overexpression of ACE2 and causes RAAS dysregulation 31, manifested by hyperdynamic circulation and elevated blood pressure, which may be associated with an increased density and activity of β-adrenergic receptors in the renal cortex. Plasma renin activity, angiotensinogen, and aldosterone levels are directly related to thyroid hormone concentration 27, 30, which regulates water and electrolyte balance through its influence on RAAS components. Several animal experiments have found that hypothyroidism increases urine volume and sodium excretion, and affects the kidneys' ability to concentrate urine, whereas hyperthyroidism tends to retain sodium 31, 32.

The expression of ACE2 in the thyroid gland has been demonstrated, and the transmembrane protease serine 2 (TMPRSS2) is also highly expressed in the thyroid, regardless of gender differences 33. David Tak Wai Lui *et al.* found that approximately 15% of patients with mild to moderate COVID-19 had thyroid dysfunction 34. This means that after COVID-19 infection, the thyroid gland is vulnerable to damage and dysfunction. Subclinical hypothyroidism is the most common in COVID-19-infected patients. In addition to the impact on the RAAS system, these patients are also more likely to develop complications such as infection. Although the improvement of subclinical hypothyroidism may help reduce complications, the effect on electrolytes remains to be elucidated. Studies have found that T3 can reduce the number of SARS-CoV-2 binding sites on the surface of target cells compared with free thyroxine (FT4) 36. However, the systemic administration of L-T3 remains controversial, and we expect to find more positive studies in the future. Most studies on COVID-19 and thyroid dysfunction have been retrospective, which makes it challenging to observe the dynamic evolution of thyroid function during COVID-19 or correlate its impact on the RAAS. The results of more prospective studies are expected to explore the effects of SARS-CoV-2 on RAAS in the pituitary-thyroid axis.

## Pituitary-adrenal axis and RAAS

**Figure 2:**
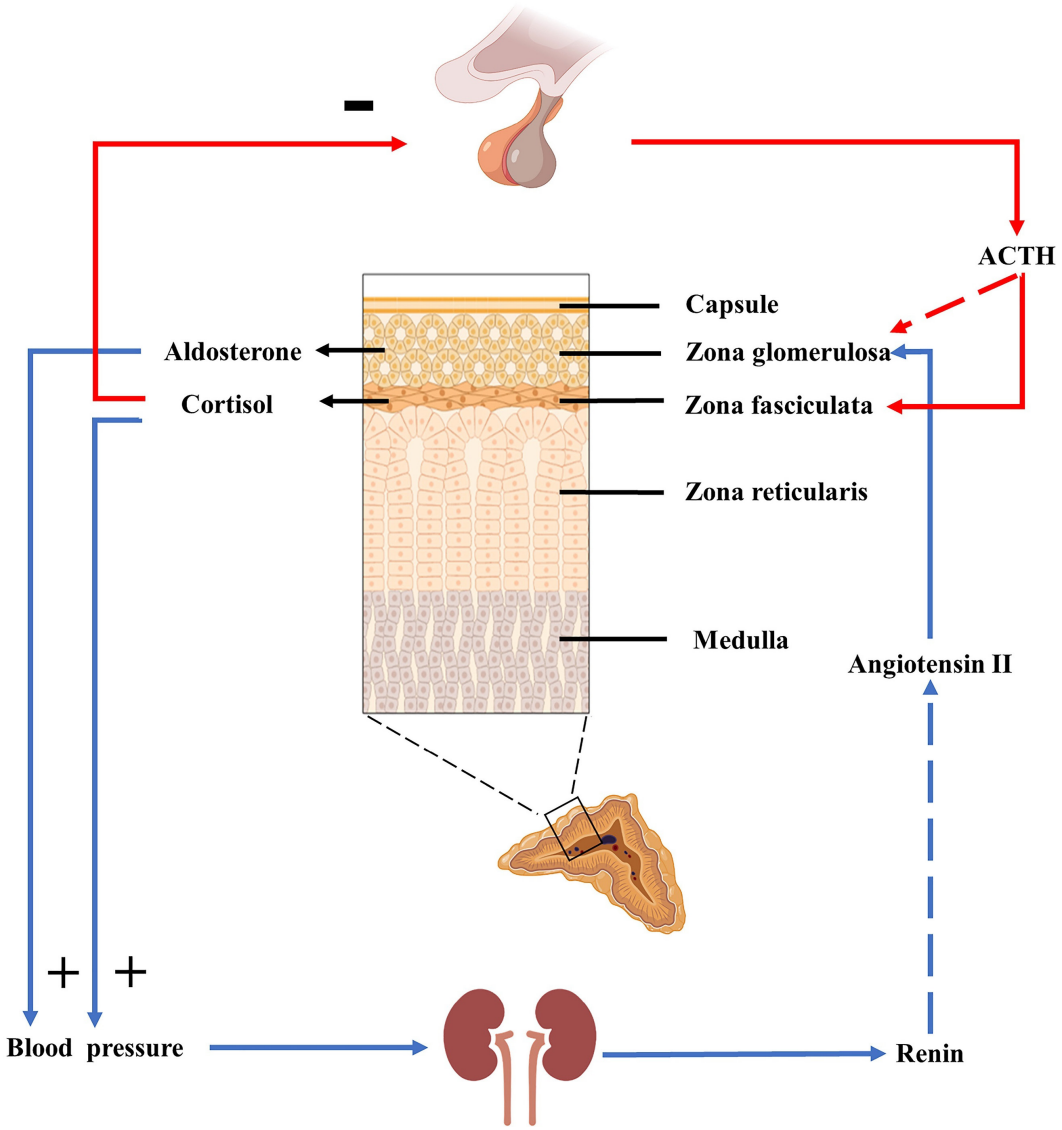
** HPA axis and RAAS.** ACTH plays a significant role in the HPA axis, promoting cortisol secretion and affecting blood pressure and RAAS. ACTH can also stimulate aldosterone secretion acutely and briefly, but this stimulation is minimal under physiological conditions.

The hypothalamic-pituitary-adrenal axis (HPA axis) is an important neuroendocrine system that mediates stress and stress-related responses, and cortisol is the primary hormone produced by the HPA axis. Under stress induction, corticotropin releasing hormone (CRH) secreted by the hypothalamus activates the pituitary gland. Then the pituitary gland releases the ACTH into the circulation (**Figure 2**). Cortisol can increase Ang II signaling in VSMC through glucocorticoid receptor-α (GRα). Ang II stimulates cortisol signaling by increasing GRα and 11β-hydroxysteroid dehydrogenase 1(11β-HSD1), and the interaction between them is conducive to the development of atherosclerosis 35. The HPA axis and RAAS are associated with increased blood pressure. A study on the Japanese population revealed that the HPA axis has a significant effect on blood pressure than the RAAS 36. Under pathological conditions, such as in patients with Cushing's syndrome, excess cortisol increases renal blood flow and sodium reabsorption in the renal tubules. Cortisol can activate Na^+^/H^+^ exchanger 3 (NHE3) in the proximal tubule 37, Na^+^-K^+^-Cl^-^ cotransporter 2 (NKCC2) in the thick ascending limb of the loop 38, Na^+^-Cl^-^ cotransporter (NCC) in the distal convolve tubule, and epithelial sodium channels (ENaC) in the collecting duct 37. In addition, cortisol can also increase the activity of the basolateral Na^+^-K^+^-ATPase 39. Excess cortisol inhibits cortisol inactivation by 11β-hydroxysteroid dehydrogenase 2(11β-HSD2), activating mineralocorticoid receptors (MR) and subsequently activating NCC and ENaC. These changes increase the kidneys' reabsorption of sodium and water, thus raising blood pressure. The physiological effects of cortisol on blood pressure and electrolytes may also be related to these changes.

**Figure 3:**
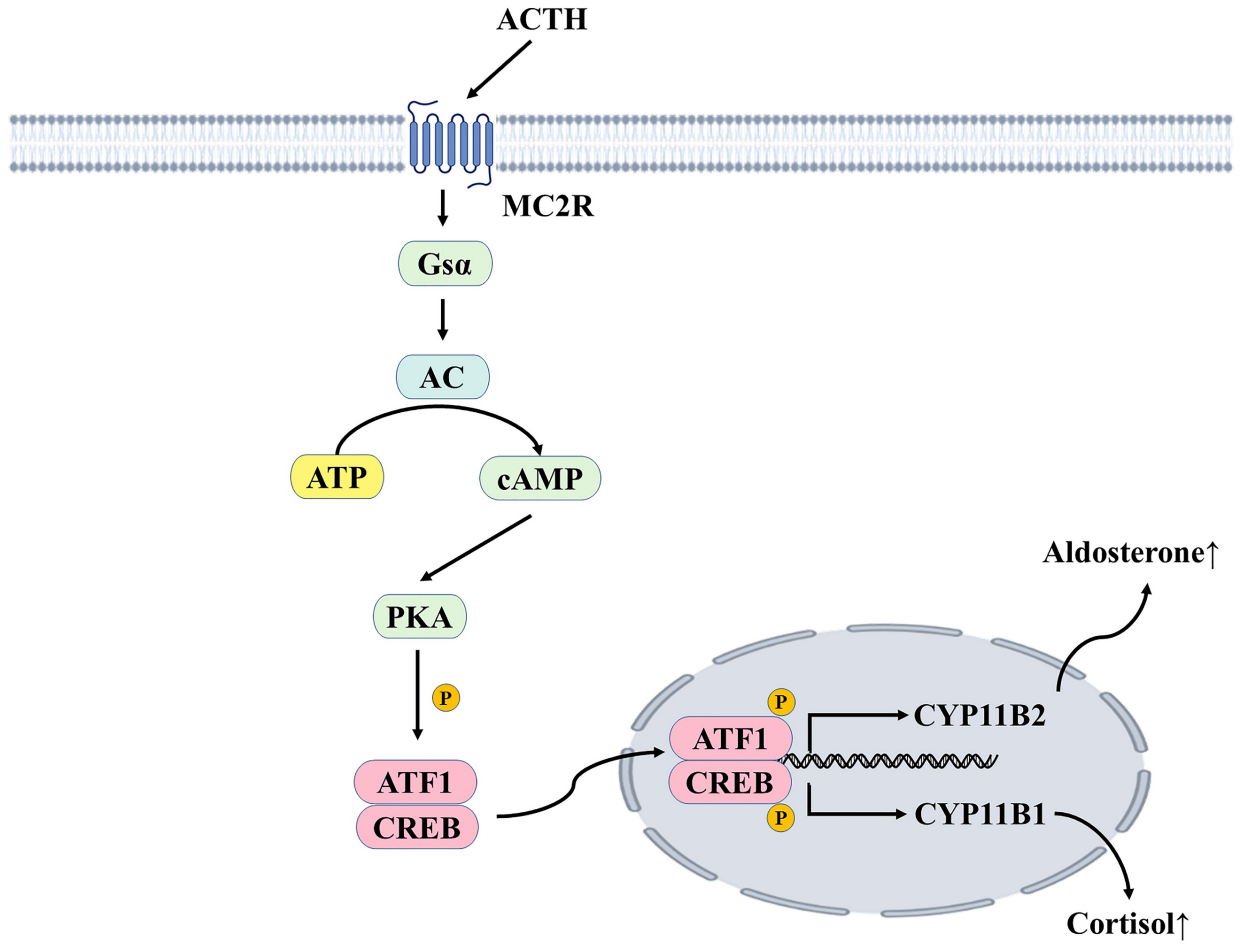
** ACTH mediates the secretion of cortisol and aldosterone.** ACTH binds to MC2R, a G-protein coupled receptor coupled with the Gsα subunit. It can increase intracellular cAMP concentration, thus activating PKA, phosphorylating CREB and ATF1, increasing the transcription of CYP11B2 and CYP11B1, and generating aldosterone and cortisol, respectively. ACTH, adrenocorticotropic hormone; MC2R, melanocortin 2 receptor; Gsα, Gs protein alpha subunit; AC, adenylate cyclase; ATP, adenosine triphosphate; cAMP, cyclic adenosine monophosphate; PKA, protein kinase A; CREB, cAMP response element binding protein; ATF1, activating transcription factor 1; P, phosphorylation.

ACTH stimulates the adrenal cortex to promote the production of cortisol in the zona fasciculata and aldosterone in the zona glomerulosa 40. ACTH binds to the melanocortin 2 receptor (MC2R) and causes stimulated adenylate cyclase to convert ATP to cyclic adenosine monophosphate(cAMP), increasing cAMP levels and stimulating protein kinase A (PKA). PKAs increase calcium influx to increase intracellular calcium levels and activate calmodulin-dependent protein kinase (CaMK). PKA and CaMK phosphorylate and activate ATF/CREB transcription factors to induce steroidogenic acute regulatory protein (StAR) and aldosterone synthase gene (CYP11B2) expression, which are early and late rate-limiting steps in aldosterone biosynthesis (**Figure 3**) 41. Aldosterone is produced primarily in the zone glomerulosa of the adrenal glands and regulates sodium and potassium balance and blood pressure by binding to MR in the kidneys. It is mainly regulated by plasma potassium and RAAS; other acute regulators include ACTH and serotonin. However, ACTH can only stimulate aldosterone secretion acutely and temporarily, to a much lower degree than the RAAS and plasma potassium 42. Aldosterone is highly responsive to the infusion of physiological doses of ACTH. The responsiveness of renin to ACTH infusion remains uncertain, with renin levels found to be either unaltered by ACTH stimulation or to exhibit delayed elevation several hours after ACTH infusion 43. However, a recent study found that increased ACTH during the stressful state was associated with higher aldosterone and renin responsiveness in acute stress-induced RAAS-related changes 44.

Both the HPA axis and RAAS play an indispensable role in the body. ACTH mainly promotes the secretion of cortisol, which mainly binds to the glucocorticoid receptor (GR), but also binds to MR. The effect of this mineralocorticoidal effect of cortisol on RAAS is often ignored. Both glucocorticoids (cortisol) and mineralocorticoids (aldosterone) regulate blood pressure and are present in disorders such as Cushing's syndrome and primary aldosteronism to contribute to hypertension. When adrenal insufficiency occurs after SARS-CoV-2 infection, it is difficult to say whether it is due to viral infection or glucocorticoid treatment. Although it has been confirmed that ACE2 and TMPRSS2 are co-localized in adrenocortical cells 45, there is currently no evidence that adrenocortical function is continuously affected 46. However, TMPRSS2 expression can be detected in the adrenal zona glomerulosa, which means that aldosterone secretion can also be affected, but little attention has been paid to the effect of SARS-CoV-2 infection on adrenocortical hormones. The HPA axis is the most studied in Long COVID syndromes, with some studies reporting that long-term impairment leads to mild and subclinical forms of central adrenal insufficiency 47. However, there is a lack of studies on the interaction between the HPA axis and RAAS in blood pressure, electrolyte regulation, and lipid metabolism, and even less on the crosstalk between the two systems after SARS-CoV-2 infection. We hope this review will inspire new ideas about these two systems.

## Pituitary-gonadal axis and RAAS

The hypothalamus produces gonadotropin-releasing hormone (GnRH), which acts on anterior pituitary gonadotrophs through the pituitary portal circulation to produce follicle stimulating hormone (FSH) and luteinizing hormone (LH). FSH mediates various biological effects through the FSH receptor (FSHR). Zhen Yu *et al.* found that FSH could promote renin production through FSHR expressed in juxtaglomerular cells, triggering the MEK/Erk and Akt signaling pathways via Gsα activation 48. LH is involved in the maturation of primordial germ cells in both sexes. LH exerts related physiological effects by binding to LH receptors, which are mainly expressed in gonadal cells. LH can promote the synthesis of prorenin in the corpus luteum. Studies have shown a direct relationship between the number of corpus luteum and the level of renin 49. However, few studies have shown the direct effects of FSH and LH on RAAS. Therefore, this part will analyze the effects of estrogen, progesterone, and androgen on RAAS from the perspective of the pituitary-gonadal axis.

### Estrogen

Estrogen is a steroid hormone associated with the female reproductive organs and is responsible for developing female sexual characteristics. Estrogen is usually divided into estrone, estradiol, and estriol. Estradiol, also known as 17-β-estradiol, is the most abundant form of estrogen in the human body, and the other two natural forms are present in lower amounts 50. A woman's circulating estrogen level fluctuates monthly and decreases during pregnancy and after menopause. Estrogen is a vasodilator, and normal estrogen levels can antagonize the RAAS by down-regulating the level of ACE and increasing the release of bradykinin, which prevents vasoconstriction and hypertension by promoting vasodilation and inducing the production of protective nitric oxide 51.

**Figure 4:**
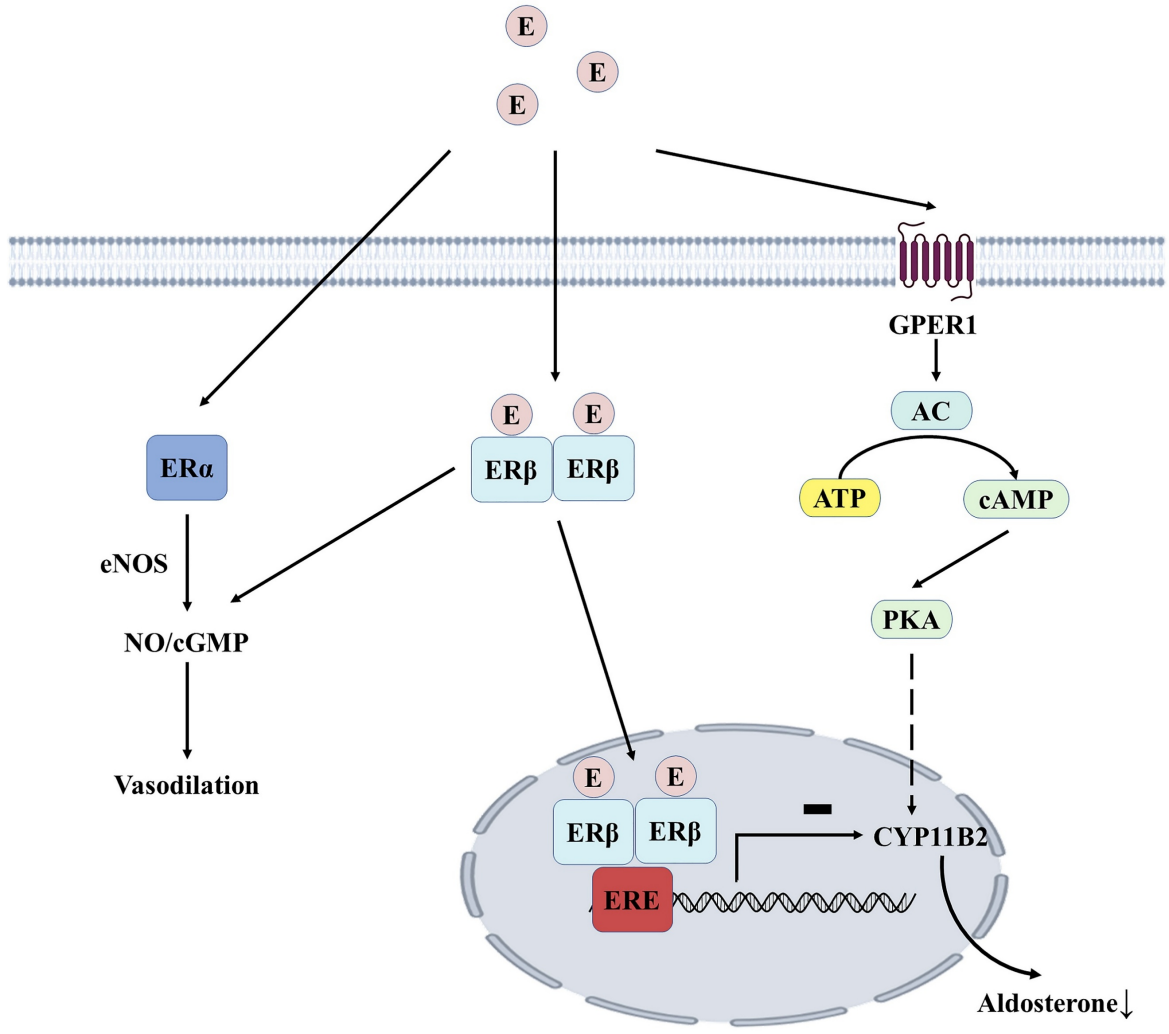
** Estrogen-mediated aldosterone production in the adrenocortical zona.** Estrogen mediates vascular endothelial cell relaxation by acting on ERα and ERβ. Estrogen dimerizes with ERβ and binds to ERE in the nucleus, inhibiting the transcription of CYP11B2, thereby reducing aldosterone synthesis. GPER1 induces the expression of aldosterone synthase through the AC/PKA signaling pathway, which ERβ-mediated effects can physiologically inhibit. E, estrogen; ERα, estrogen receptor type α; ERβ, estrogen receptor type β; GPER1, G protein-coupled estrogen receptor type 1; eNOS, nitric oxide synthase; NO, nitric oxide;cGMP, cyclic guanosine monophosphate; ERE, estrogen response element; AC, adenylate cyclase; ATP, adenosine triphosphate; cAMP, cyclic adenosine monophosphate; PKA, protein kinase A.

Estrogen can bind to estrogen receptors (ERα and ERβ) in endothelial and vascular smooth muscle cells, increasing NO bioavailability and promoting vasodilation. For example, 17β-estradiol can delay the aging of vascular endothelial cells (VECs) by acting on ERα 52. ERβ can mediate vascular relaxation, and the deficiency of this receptor can lead to vascular dysfunction and hypertension 53. The adrenal cortex expresses all estrogen receptor subtypes, with the highest expression of ERβ in the zona glomerulosa. In premenopausal women, high estrogen levels may inhibit aldosterone synthesis through ERβ, which could explain why estrogen hormone replacement therapy reduces blood pressure in postmenopausal women (**Figure 4**). After menopause, the antihypertensive effect of estrogen-mediated by ERβ disappears. In contrast, the release of aldosterone mediated by G protein-coupled estrogen receptor 1(GPER1) promotes increased blood pressure, partly explaining the high prevalence of resistant hypertension in postmenopausal women 54. Overactivation of RAAS has been implicated in the pathogenesis of hypertension, and both endogenous and exogenous estrogens alter the balance between vasoconstriction and relaxation of RAAS.

There are gender differences in RAAS. The expression level of ACE/Ang-II/AT1R axis is higher in males, while ACE2/Ang(1-7)/Mas is more active in females, which means that RAAS in males is more susceptible to SARS-CoV-2 and has a higher risk of serious complications and death after illness 55. The down-regulation of ACE2 by SARS-CoV-2 leads to increased Ang II, which in turn causes vasoconstriction, hypertension, and inflammation. 17β-estradiol can enhance the expression of ACE2 (ACE2/Mas axis) and reduce ARDS by controlling RAAS 56. Estrogen may also exert a greater protective effect on the vasculature by activating endothelial nitric oxide synthase (eNOS) and stimulating NO and cyclic guanosine monophosphate (cGMP). COVID-19 severity was reduced in postmenopausal women who received supplemental estrogen compared to age-matched women and men who did not take estrogen 57. The potential protective effect of estradiol was further illustrated by the study by Ute Seeland *et al.*, which found that women receiving treatment containing estradiol had a 50% lower risk of death after SARS-CoV-2 infection compared with premenopausal women not receiving hormone replacement therapy (HRT) 58. 17β-estradiol levels decline with age in postmenopausal women, making women more susceptible to RAAS-related conditions such as ARDS and other acute lung injury diseases, hypertension, and cardiovascular disease. Estrogen therapy may provide a new direction for the treatment of SARS-CoV-2 infection.

### Progesterone

Progesterone is an endogenous steroid hormone typically produced by the adrenal cortex, ovaries, and testes, and its primary role is maintaining pregnancy. Progesterone has a neutral or inhibitory effect on blood pressure 59, and the decrease in blood pressure is positively correlated with the increase in progesterone as pregnancy progresses 60. Several studies have found that similar to estradiol, progesterone induces endothelium-dependent vasodilation 61.

Progesterone has the potential to affect aldosterone production through several mechanisms. Progesterone is a competitive inhibitor of MRs, and progestin-induced urinary sodium excretion may lead to compensatory activation of the RAS in the luteal phase 62, 63. Secondary progestin-induced vasodilation could also lead to RAS activation 64, and PRA and AngII would be expected to increase in parallel with aldosterone levels if this were the primary mechanism at work. However, in actual clinical studies, exogenous administration of progesterone did not find an increase in plasma renin activity and AngII 65-67. Emily D Szmuilowicz *et al.* added progesterone to isolated rat adrenal zona glomerulosa cells, resulting in a 2.8-fold increase in aldosterone production. In contrast, the addition of estradiol had no effect. Suggesting that progesterone may directly affect adrenal aldosterone production, which may also be a potential mechanism for increased aldosterone production in a physiological state of high progesterone 67.

There is some evidence that progesterone may also show sex differences in the treatment of SARS-CoV-2 infection, and Ghandehari *et al.* 68 used progesterone (100 mg, subcutaneously, twice a day, for up to 5 days) in a randomized controlled trial to treat hospitalized male patients with moderate to severe COVID-19 and found improvement in hypoxemia in male hospitalized patients. Electrolyte disturbances secondary to SARS-CoV-2 infection are common 69, and Elabida *et al.* have provided strong evidence for the important role of progesterone in regulating electrolyte balance 70. Estrogen and progestin as complementary therapy after SARS-CoV-2 infection is currently being evaluated in multiple studies 71, and exciting trial results are expected.

### Androgen

**Figure 5:**
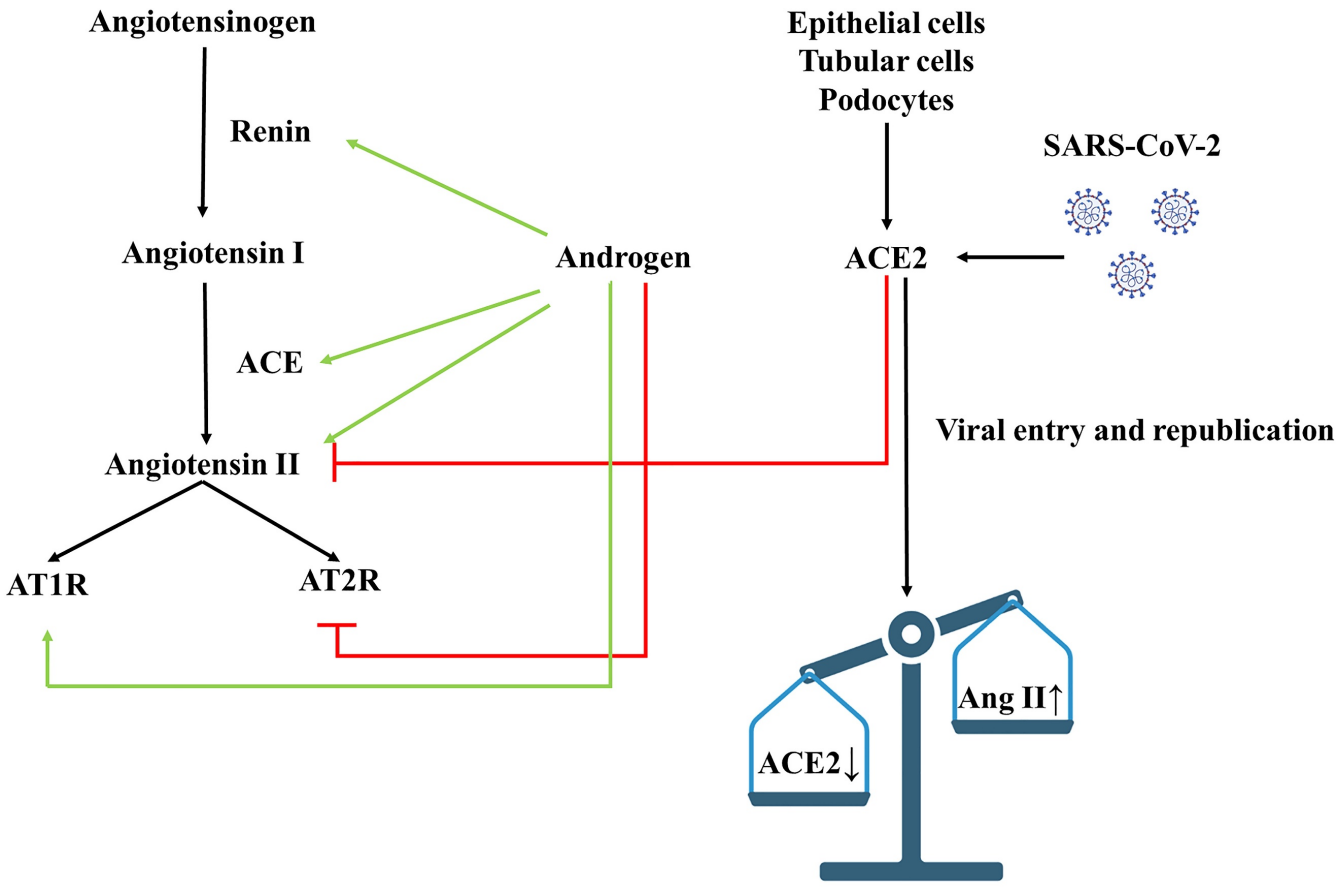
** Effects of androgen and SARS-CoV-2 on RAAS. Androgens increase blood pressure by affecting RAAS-related components, and ACE2-mediated SARS-CoV-2 infection causes acute kidney damage.** The red line represents inhibition, the green line represents facilitation, and the black line represents downstream influence. ACE, angiotensin-converting enzyme; ACE2, angiotensin-converting enzyme 2; AT1R, angiotensin II receptor type 1; AT2R, angiotensin II receptor type 2; AngII, angiotensin II.

Androgens are endocrine hormones that maintain reproduction and metabolism. Testosterone and its active metabolite dihydrotestosterone (DHT) are the major androgens in the circulation of mature male mammals. "Classical androgen signaling" refers to the genomic effects mediated by the androgen receptor (AR) in the cytoplasm 72. Testosterone and DHT are ligands for AR. Testosterone increases renin levels, ACE, and AT1R expression, while down-regulating AT2R expression, leading to vasoconstriction 73, 74. Androgen has been found to enhance vascular responsiveness to Ang II in hypertensive rats 75. By studying the relationship between testosterone and blood pressure, Tina Kienitz *et al.* 76 found that androgen signaling stimulates Na^+^ reabsorption in the kidney's proximal tubule, which may be related to the up-regulation of intrarenal Ang II. Secondly, the interaction between testosterone and ARs, up-regulating αENaC expression by serum/glucocorticoid regulated kinase 1 (SGK1) and blocking the endocytosis of ENaC mediated by Nedd4-2 phosphorylation, may be important in developing hypertension. In a mouse model of polycystic ovary syndrome, long-term chronic administration of DHT increased angiotensinogen mRNA expression by 9-fold and ACE mRNA by approximately 0.5-fold 77. Numerous studies have shown 78, 79 that testosterone may shift the balance of RAS toward Ang II-ACE-AT1R pathway to induce vasoconstriction, vascular dysfunction, and elevated blood pressure (**Figure 5**).

SARS-CoV-2 enters cells by binding to ACE2 and TMPRSS2, which is also expressed in adult Leydig cells 80, so infection with SARS-CoV-2 may alter the ability of the testis to produce or secrete androgens 81. Testosterone may act as a double-edged sword in the SARS-CoV-2 pandemic. On the one hand, androgens can affect the immune response by increasing cytokine production and decreasing antibody responses to infectious diseases, which can lead to severe infections in men 82. On the other hand, some studies have found that in elderly men, lower testosterone levels reduce immunosuppression, making them more likely to develop a solid immune inflammatory response, and the degree of testosterone reduction is a predictor of disease severity after SARS-CoV-2 infection 83, 84. This indicates that androgens can be used as adjuvant therapy in patients with COVID-19, as an earlier study demonstrates 85. However, the severity of lung involvement during COVID-19 infection is associated with androgen excess 86. Indeed, androgen-modulating drugs have been proposed as potential treatments for COVID-19^87^, and prostate cancer patients receiving antiandrogen therapy appear to be partially protected from SARS-CoV-2 infection 88. The short- or long-term effects of SARS-CoV-2 infection are still unclear, and the role of low and high levels of testosterone remains controversial 90. Therefore, we look forward to more comprehensive studies to explore the role of SARS-CoV-2 infection in testosterone and blood pressure.

### Summary

The pituitary-gonadal axis affects RAAS by affecting the secretion of various sex hormones, thereby regulating the water and sodium balance. It suggests that we should not ignore the changes and potential role of gonadal hormones in the disease's cognition and related hormone replacement therapy. Some studies have found that SARS-CoV-2 infection impacts the HPG axis. The current evidence has only partially explained the role of sex hormones in COVID-19. The use of hormone replacement or suppression therapy is still controversial, and different sex hormone therapy has different benefits and risks after SARS-CoV-2 infection (**Table 1**). However, the long-term follow-up data on the level of sex-related hormones before and after SARS-CoV-2 infection is not perfect, and the potential impact on the HPG axis cannot be analyzed comprehensively.

## Pituitary-growth hormone axis and RAAS

Growth hormone (GH) is a protein secreted by growth hormone cells in the anterior pituitary gland. Its primary function is to promote the growth and development of children and adolescents, but it also plays other vital roles in whole-life activities, such as affecting the metabolism of glucose, fats, and proteins. Other potential effects, such as those on fibrosis, cardiovascular function, and cancer, are also being recognized 89, 90. GH produces a direct or indirect effect, the direct effect is achieved by binding to GH receptor (GHR) on target cells, and the indirect effect is that GH acts on the liver to stimulate the synthesis and secretion of insulin-like growth factor-1 (IGF-1), and then affects the growth and metabolism of surrounding tissues 91.

**Figure 6:**
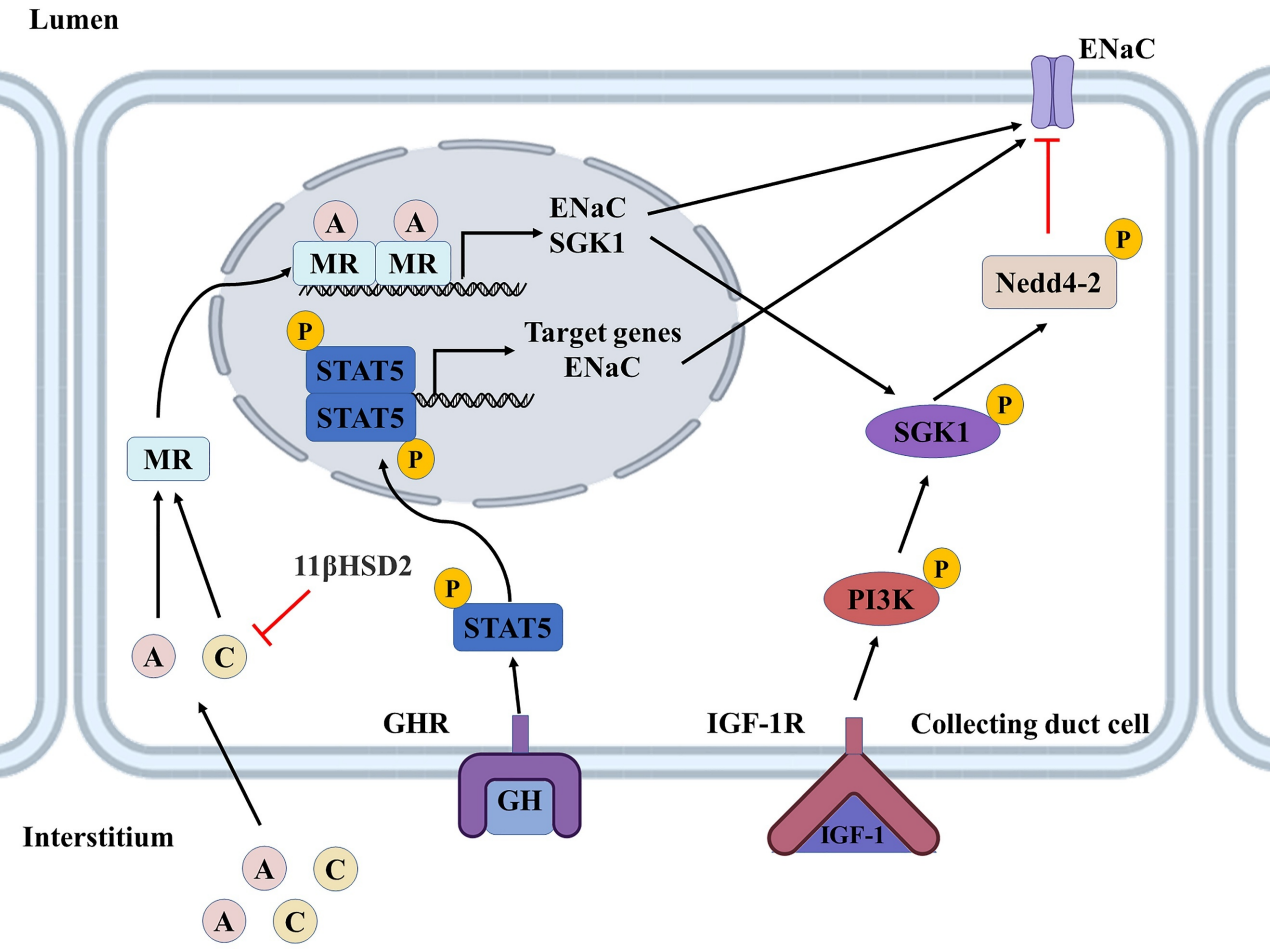
** Role of GH/IGF-1, aldosterone, and cortisol in renal collecting ducts.** GH binding to GHR triggers activation of the STAT5 pathway, leading to transcriptional activation of GH target genes and increased expression of ENaC. IGF-1 can be synthesized locally or captured from the circulation, bound to IGF-1R, and activated via PI3K-dependent SGK1. SGK1, phosphorylating Nedd4/2, can inhibit its transport and degradation of ENaC. These two hormones act in concert and together regulate epithelial sodium transport. Aldosterone regulates ENaC by forming a dimer with MR to enter the nucleus and promote the expression of SGK1 and ENaC. The mineralocorticoid effect of cortisol is affected by 11β-HSD2, which converts cortisol to an inactive form and protects MR from activation by cortisol. Red lines indicate inhibition by phosphorylation or ubiquitination, and black lines indicate material movement or change to a phosphorylated state. GH, growth hormone; GHR, growth hormone receptor; IGF-1, insulin-like growth factor 1; IGF-1R, insulin-like growth factor 1 receptor; STAT5,signal transducer and activator of transcription 5; PI3K, phosphoinositide 3-kinase; SGK1, serum/Glucocorticoid regulated kinase 1; Nedd4-2, neural precursor cell expressed, developmentally down-regulated 4-2; ENaC, epithelial Na+ channel; A, aldosterone; C, cortisol; MR, mineralocorticoid receptor; 11β-HSD2, 11β-hydroxysteroid dehydrogenase 2; P, phosphorylated.

The effects of GH are mediated mainly by GH-induced growth factors, among which IGF-1 has been the most widely studied in physiology 90, and GH/IGF-1 has long been considered an essential regulatory system for renal tubular sodium and water reabsorption 92. GH deficiency is associated with lower sodium and water levels in the body, which can be ameliorated by rhGH replacement therapy 93. Conversely, excessive GH in the body or treatment with high doses of rhGH may even lead to acute fluid retention 94. Recent studies find the pituitary-growth hormone axis for sodium and water reabsorption in renal tubules via IGF-1, considering that both GH and IGF-1 receptors are expressed throughout the nephron, the site of GH/IGF-1 regulation of sodium reabsorption has been controversial. One of the most apparent effects of GH and IGF-1 on the kidney is their role in sodium retention in the distal tubule 95. In cortical collecting duct cells, GH binding to GHR triggers activation of the JAK2/STAT5 and MAPK pathways. This results in transcriptional activation of kidney-specific GH target genes, including αENaC. IGF-1, locally synthesized in the kidney or obtained from the peripheral circulation, binds to IGF-1R and is activated by phosphatidylinositol 3-kinase (PI3K) -dependent SGK1, whose phosphorylation inhibits the ubiquitin ligase Nedd4-2, thereby regulating the expression of ENaC on the cell membrane surface 96. These two mechanisms synergistically promote sodium reabsorption in the collecting ducts (**Figure 6**) 97.

Our previous study suggested that GH/IGF-1 could regulate the components of RAAS, promote sodium and water reabsorption, induce the increase of extracellular fluid, and even contribute to hypertension. Overexpression of GH can inhibit the ACE2/Ang-(1-7)/Mas axis, a finding confirmed in relevant animal experiments 98, 99. Interestingly, following SARS-CoV-2 infection, several studies have found a possible association between low IGF-1 levels (and possibly GH) and poor outcomes in patients with COVID-19 101, 102, while a lower rate of positive SARS-CoV-2 infection was observed in children receiving GH replacement therapy 100. It seems to be somewhat contradictory to our current knowledge, as some studies have found that high expression of IGF-1 reduces COVID-19 susceptibility as well as poor prognosis 101, 102, but the resulting changes in blood pressure have not been studied in great intensity because hypertension is a risk factor for COVID-19 disease 103. GH replacement therapy has played a positive role in children with SARS-CoV-2 infection, but it has side effects such as fluid retention, peripheral edema, arthralgia, and the risk of cancer in adults, especially elderly patients 104. It suggests that we need to better understand the effects of GH/IGF-1 on RAAS under physiological and pathological conditions and the cascade of effects, which will help us understand the role of GH/IGF-1 in COVID-19.

## Arginine vasopressin (AVP) and RAAS

Antidiuretic hormone (ADH) or arginine vasopressin (AVP) is a nonapeptide synthesized in the supraoptic nucleus (SON) and paraventricular nucleus (PVN) of the hypothalamus and stored in the posterior pituitary gland. It is vital in regulating blood pressure, maintaining water and sodium balance, and maintaining renal function 105. There are three distinct vasopressin receptor (VR) subtypes, V1aR, V1bR, and V2R. V1aR is mainly expressed in vascular smooth muscle to cause vasoconstriction. It also mediates cell proliferation, platelet aggregation, glycogenolysis, and lipid metabolism 106, 107. V1bR is expressed in anterior pituitary cells and mainly stimulates the release of ACTH 108. V2R mainly exists in the thick ascending limbs of Henle's loop (TAL) and collecting duct and promotes the expression of aquaporin 2 (AQP2) to increase water reabsorption 109.

**Figure 7:**
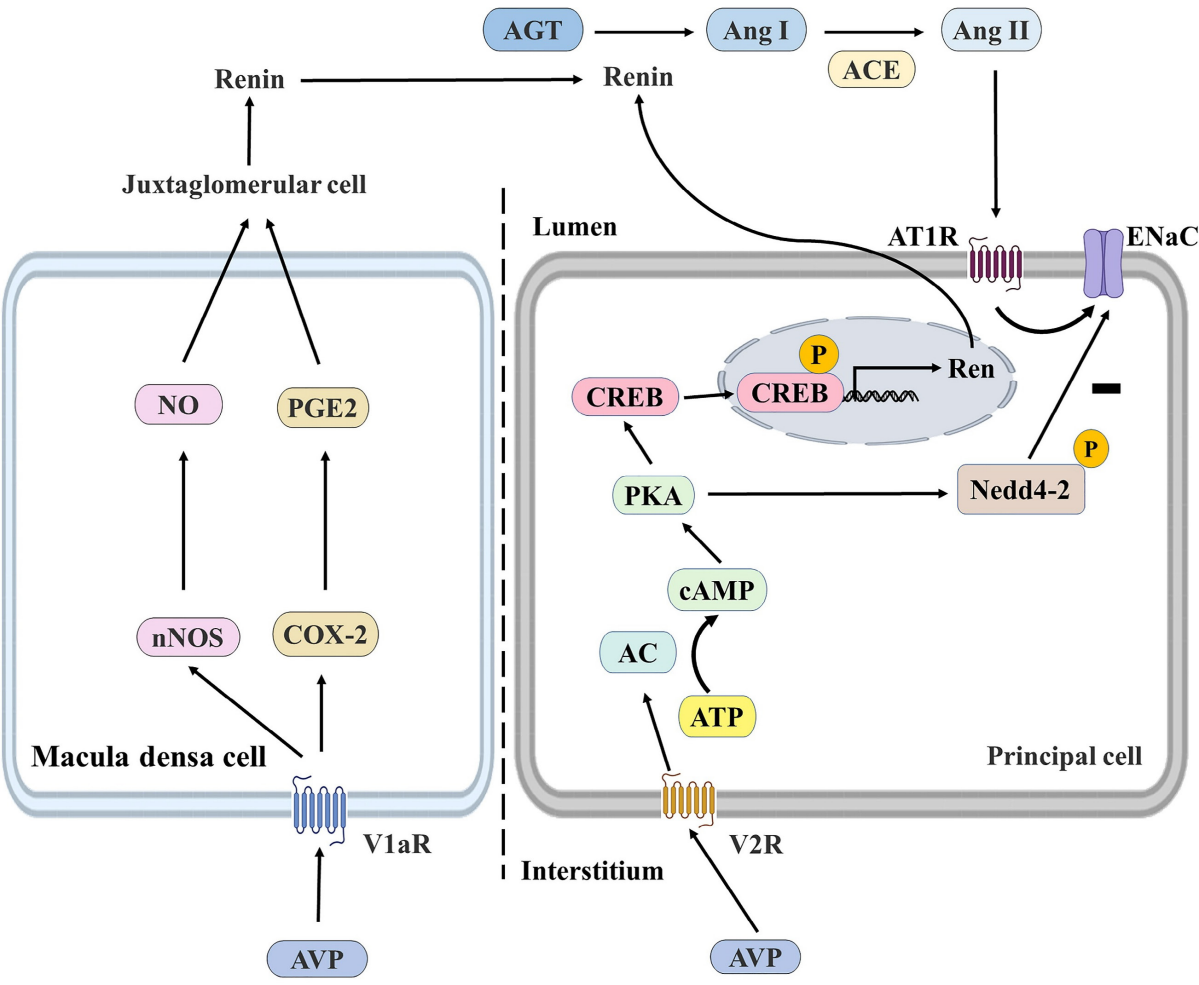
** The regulation of renin in the macula densa and collecting ducts.** AVP can bind to the V1aR of macula densa cells and the V2R of principal cells. AVP/V1aR stimulates the expression of nNOS and COX-2, leading to NO and PGE2 production by macula densa cells, which stimulates renin production by juxtaglomerular cells and subsequently induces an increase in Ang II and aldosterone levels to promote water reabsorption. AVP/V2R promotes renin gene expression by enhancing CREB phosphorylation through the cAMP/PKA pathway, and PKA also reduces ENaC degradation by phosphorylating Nedd4-2. AVP, arginine vasopressin; nNOS, neuronal nitric oxide synthase; COX-2, cyclooxygenase-2; NO, nitric oxide; PGE2, prostaglandin E2; AGT, angiotensinogen; Ang I, angiotensin I; Ang II, angiotensin II; ACE, angiotensin-converting enzyme; AC, adenylate cyclase; ATP, adenosine triphosphate; cAMP, cyclic adenosine monophosphate; PKA, protein kinase A; CREB, cAMP response element binding protein; V1aR, vasopressin receptor type 1a; V2R, vasopressin receptor type 2; AT1R, angiotensin II receptor type 1; ENaC, epithelial Na+ channel; Nedd4-2, neural precursor cell expressed, developmentally down-regulated 4-2; P, phosphorylated.

In this part, we mainly discussed the functional crosstalk between AVP and RAAS and the influence of AVPs on water and sodium balance. It was found that AVP had specific effects on RAAS by increasing ENaC activity and binding with V1aR expressed by macula densa cells. ENaC is expressed in the apical cell membrane of aldosterone-sensitive distal nephritis (ASDN) 110, and aldosterone stimulates ENaC in ASDN via MR to minimize renal sodium excretion. V1aR has also been detected in the juxtaglomerular apparatus, the thick ascending limbs of Henle's loop, and collecting ducts of the kidney 111, 112. AVP may also bind to V1aR in macula densa cells, inducing renin secretion, triggering RAAS activity, and indirectly increasing sodium reabsorption 113, 114. Toshinori Aoyagi *et al.* demonstrated the effect of AVP/V1aR on renin secretion. They found that renin and Ang II were reduced in V1aR-deficient (V1aR^-/-^) mice. It was also found that the expression of neuronal nitric oxide synthase and cyclooxygenase-2 was decreased in V1aR-specific macula densa cells, suggesting that AVP regulates the V2R-AQP2 system and glomerular filtration rate by stimulating the V1aR in the glomerular macula densa cells and thereby activating RAAS 114. Secondly, Alexis A. Gonzalez *et al.* showed that V2R activation in mouse renal collecting duct cell lines (M-1 cell cultures) increased renin synthesis and secretion, and the V2R antagonist tolvaptan could block this effect and further demonstrated that the V2R-PKA- CREB signaling pathway in collecting duct renin production, which is independent of Ang II/AT1R (**Figure 7**) 115.

SARS-CoV-2 infection induces ACE2 downregulation, reducing vasodilator and protective Ang 1-7 and accumulating Ang II. Elevated Ang II stimulates the release of AVPs in the hypothalamus, leading to hyponatremia and the release of inflammatory cytokines 116. Secondly, activated immune cells (mainly T and B lymphocytes) and the release of proinflammatory cytokines stimulate immune cells to release stored AVP, and this vicious cycle further triggers the release of proinflammatory cytokines 117. Acute injury caused by SARS-CoV-2 infection induces the non-osmotic release of AVPs, leading to the syndrome of inappropriate antidiuresis (SIADH) and increased release of AVPs 118. Many studies have shown that hyponatremia is associated with complications and mortality in COVID-19 patients 119, 120, and AVP receptor antagonists (VRAs) have also been shown to be a potential treatment for COVID-19 by alleviating AVP-mediated hyponatremia 121.

## Conclusion

Hormones produced by the pituitary gland, such as TSH, ACTH, LH/FSH, GH, and AVP, affect the balance of RAAS through their direct or indirect effects. In endocrine diseases and many diseases involving water and sodium regulation and blood volume change, we should think about the clinical manifestations and treatment of diseases from more directions in order to obtain greater clinical benefits. In the post-pandemic era, the effects of SARS-CoV-2 have not entirely dissipated, and we should conduct long-term follow-up, observation, and research on the adverse effects of SARS-CoV-2 infection.

## Figures and Tables

**Table 1:** Benefits and risks of different hormone therapies in patients with SARS-CoV-2 infection

	Benefits	Risks
Estrogen therapy	Anti-inflammatory effects 122-124 Endothelial protection and vasodilation 125 Immunoresistance and immunomodulation 124, 126-128 Antioxidant 129 Antiviral effects 130, 131	Thrombosis 132, 133 Impaired glucose regulation 134, 135
Progesterone therapy	Regulation of immunity 136, 137 Anti-inflammatory effects 138-140 Antiviral effects 128, 141, 142	
Antiandrogen therapy	Regulation of immunity 143-145 Reducing the risk of thrombosis 146, 147 Reduced mortality and hospitalization rates 148, 149	Increased infection 150
